# Development of PSMA-PET-guided CT-based radiomic signature to predict biochemical recurrence after salvage radiotherapy

**DOI:** 10.1007/s00259-023-06195-3

**Published:** 2023-03-16

**Authors:** Simon K. B. Spohn, Nina-Sophie Schmidt-Hegemann, Juri Ruf, Michael Mix, Matthias Benndorf, Fabian Bamberg, Marcus R. Makowski, Simon Kirste, Alexander Rühle, Jerome Nouvel, Tanja Sprave, Marco M. E. Vogel, Polina Galitsnaya, Jürgen E. Gschwend, Christian Gratzke, Christian Stief, Steffen Löck, Alex Zwanenburg, Christian Trapp, Denise Bernhardt, Stephan G. Nekolla, Minglun Li, Claus Belka, Stephanie E. Combs, Matthias Eiber, Lena Unterrainer, Marcus Unterrainer, Peter Bartenstein, Anca-L. Grosu, Constantinos Zamboglou, Jan C. Peeken

**Affiliations:** 1 Department of Radiation Oncology, University Medical Center Freiburg, Faculty of Medicine, University of Freiburg, Robert-Koch-Straße 3, 79106 Freiburg, Germany; 2 German Cancer Consortium (DKTK) Partner Site Freiburg, Heidelberg, Germany; 3 Berta-Ottenstein-Programme, Faculty of Medicine, University of Freiburg, Freiburg, Germany; 4 Department of Radiation Oncology, University Hospital, LMU Munich, Munich, Germany; 5 Department of Nuclear Medicine, Faculty of Medicine, Medical Center, University of Freiburg, Freiburg, Germany; 6 Department of Radiology, University Medical Center Freiburg, Faculty of Medicine, University of Freiburg, Freiburg, Germany; 7 Department of Radiology, Klinikum rechts der Isar, Technical University of Munich, Munich, Germany; 8 Department of Radiation Oncology, Klinikum rechts der Isar, Technical University of Munich, Munich, Germany; 9 German Cancer Consortium (DKTK), Partner Site Munich, Munich, Germany; 10 Department of Urology, Klinikum rechts der Isar, Technical University of Munich, Munich, Germany; 11 Department of Urology, University Medical Center Freiburg, Faculty of Medicine, University of Freiburg, Freiburg, Germany; 12 Department of Urology, University Hospital, LMU Munich, Munich, Germany; 13 OncoRay - National Center for Radiation Research in Oncology, Faculty of Medicine and University Hospital Carl Gustav Carus, Technische Universität Dresden, Helmholtz-Zentrum Dresden - Rossendorf, Dresden, Germany; 14 National Center for Tumor Diseases (NCT), Partner Site Dresden, Dresden, Germany; 15 German Cancer Consortium (DKTK) Partner Site Dresden, Heidelberg, Germany; 16 Faculty of Medicine and University Hospital Carl Gustav Carus, Technische Universität Dresden, Dresden, Germany; 17 Helmholtz Association/Helmholtz-Zentrum Dresden - Rossendorf (HZDR), Dresden, Germany; 18 Department of Nuclear Medicine, Klinikum rechts der Isar, Technical University of Munich, Munich, Germany; 19 Institute of Radiation Medicine, Helmholtz Zentrum München, Munich, Germany; 20 Department of Nuclear Medicine, University Hospital, LMU Munich, Munich, Germany; 21 German Oncology Center, European University of Cyprus, Limassol, Cyprus

**Keywords:** PSMA-PET/CT, Salvage radiotherapy, Prostate cancer, Radiomics, Outcome prediction, Personalization

## Abstract

**Purpose:**

To develop a CT-based radiomic signature to predict biochemical recurrence (BCR) in prostate cancer patients after sRT guided by positron-emission tomography targeting prostate-specific membrane antigen (PSMA-PET).

**Material and methods:**

Consecutive patients, who underwent ^68^Ga-PSMA11-PET/CT-guided sRT from three high-volume centers in Germany, were included in this retrospective multicenter study. Patients had PET-positive local recurrences and were treated with intensity-modulated sRT. Radiomic features were extracted from volumes of interests on CT guided by focal PSMA-PET uptakes. After preprocessing, clinical, radiomics, and combined clinical-radiomic models were developed combining different feature reduction techniques and Cox proportional hazard models within a nested cross validation approach.

**Results:**

Among 99 patients, median interval until BCR was the radiomic models outperformed clinical models and combined clinical-radiomic models for prediction of BCR with a C-index of 0.71 compared to 0.53 and 0.63 in the test sets, respectively. In contrast to the other models, the radiomic model achieved significantly improved patient stratification in Kaplan-Meier analysis. The radiomic and clinical-radiomic model achieved a significantly better time-dependent net reclassification improvement index (0.392 and 0.762, respectively) compared to the clinical model. Decision curve analysis demonstrated a clinical net benefit for both models. Mean intensity was the most predictive radiomic feature.

**Conclusion:**

This is the first study to develop a PSMA-PET-guided CT-based radiomic model to predict BCR after sRT. The radiomic models outperformed clinical models and might contribute to guide personalized treatment decisions.

**Supplementary Information:**

The online version contains supplementary material available at 10.1007/s00259-023-06195-3.

## Introduction

Patients who undergo radical prostatectomy (RPE) for localized prostate cancer (PCa) as initial treatment experience biochemical recurrence (BCR) in up to 50% within 5 years after treatment [1]. Adverse pathological features are associated with higher rates of BCR [2]. Salvage radiotherapy (sRT) with or without androgen deprivation therapy (ADT) provides the only curative treatment options for these patients and should be initiated at low PSA levels [3]. Since response rates are heterogeneous [4], tools for improved risk stratifications are warranted in order to identify patients who are at higher or lower risk for relapse after sRT and thus might be candidates for therapy intensification or de-intensification.

Positron-emission tomography targeting prostate-specific membrane antigen (PSMA-PET) combined with computer tomography (CT) significantly improved detection rates of local (LR) and nodal recurrence (NR) RPE [5] and altered treatment management [6], but prospective evidence of putative beneficial effects on outcomes are pending.

Despite the great diagnostic value of modern imaging technologies, the computer-based extraction and analysis of image features (radiomics) offers new opportunities for improved image analysis to provide additional information about tumor characteristics as shown for multiple cancer entities [7–10]. Various studies have reported on improvements for PCa detection, prediction of ISUP, ECE, and BCR in primary PCa patients but no data exists in the setting of sRT [11].

In order to identify novel markers for personalized risk stratification, this retrospective multicenter study aims to evaluate the impact of PSMA-PET-guided CT-based radiomic features (RF) derived from PSMA-PET/CT scans on BCR-free survival (BRFS) in patients who receive sRT due to recurrent or persistent PCa cancer after RPE.

## Material and methods

### Patients and treatment

This retrospective multicenter study pooled patients from three high-volume centers in Germany (University Medical Centre Freiburg (UKF), Klinikum Rechts der Isar Technical University Munich (TUM), University Hospital of the Ludwig-Maximillian’s-University Munich (LMU)). Each center received an institutional review board approval for this study (Freiburg No.: 15/18; TUM:466/16 S; LMU: 17-765). Written informed consent was waived due to the retrospective nature of the study.

Data from patients who received radical surgery and underwent ^68^Ga-PSMA11-PET/CT due to PSA persistence (PSA after surgery ≥ 0.1 ng/ml) or recurrence (PSA ≥ 0.2 as nadir after surgery) and were subsequently treated with PSMA-PET-guided sRT were collected. From the total cohort, only patients who received a contrast-enhanced CT were included in this analysis. Patients with distant metastases (lymph nodes above the iliac bifurcation, bone metastases, or visceral metastases) present in PSMA-PET/CT and if ADT was given prior to PSMA-PET/CT scans were excluded. Treatment decisions were taken locally at the discretion of the treating physicians according to standards of care at the time of treatment [3] and based on PSMA-PET/CT findings. See Supplemental Methods Table S1 for details on salvage RT concepts for each center. ADT was administered at the discretion of the treating physician. In total, 99 patients with PET-positive local recurrence treated with sRT between 2014 and 2020 met the inclusion criteria.

### Data collection and follow-up

The following clinical data were collected: age at sRT, International Society of Urologic Pathology Grading (ISUP), pathological T-stage and N-stage, initial PSA and PSA prior to sRT, presence of nodal recurrence, administration and duration of ADT, and sRT doses. Follow-up assessments included serum PSA testing at regular intervals based on institutional clinical standards.

### ^68^Ga-PSMA11 PET/CT

^68^Ga-PSMA11 was synthesized according to good manufacture practice in all centers and in accordance with international procedural guidelines [12]. Acquisition protocols and scanner types are provided in the Supplemental Methods.

All scanners fulfilled the requirements indicated in the European Association of Nuclear Medicine (EANM) imaging guidelines and obtained EANM Research Ltd. (EARL1) accreditation during acquisition.

See [13] for details on PSMA image acquisition and reconstruction algorithms.

All PSMA-PET/CT images were reviewed locally prior to data sharing according to reporting international guidelines [14] by two nuclear medicine physicians with experience on PCa imaging. Disagreements were resolved by consensus.

### Segmentation

Further image processing was performed using the 3D Slicer v4.10.0 [23]. Two separate segmentation strategies were followed: First, considering the local nuclear medicine report, PSMA-PET-positive PCa lesions were manually contoured within the CT image by one reader (SS) with  >3 years’ experience in PSMA-PET/CT segmentation guided by the PSMA-PET signal using validated segmentation approach levels [15]. Second, 20% of the maximal standard uptake value (SUVmax) of the lesions was used as a threshold for PET-based semi-automatic segmentations.

### Radiomic feature extraction

Radiomic feature and preprocessing were performed using the pyradiomics package (version 3.0.1) in Python (version 3.7.9) [16]. For preprocessing, a fixed bin width of 5 HU was used for image discretization [17]. Isotropic resampling was performed to a voxel size of 1 × 1 × 1 mm using Bspline interpolation. Shape, first-order, and texture features were computed from the original image according to the “image biomarker standardization initiative” guidelines [18]. Texture matrices were aggregated averaged over 3D directions for GLCM and GLRLM, or 3D for GLSZM, NGTDM, and GLDM features. See Supplemental Table S2 for a list of the total 104 features.

### Modeling strategy and statistical analyses

The modeling steps were performed using the *familiar package* (0.0.0.53) in R (version 4.1.2, R core team, Vienne, Austria) (https://github.com/alexzwanenburg/familiar). For signature building, a recently published approach was chosen [19]. See Supplemental Methods for a detailed description. In brief, the radiomic feature space was reduced by excluding features susceptible (intraclass correlation coefficient (ICC 3.1)  <0.8) to small differences in the segmentation type (manual vs. PET threshold-based) and of features highly correlated with clinical variables (PSA initial, ISUP, rcN, max PSA) (Spearman coefficient  ≤0.6). Third, we performed hierarchical clustering with complete linkage and Spearman correlation as a distance metric keeping one representative feature from each cluster.

Finally, Cox proportional hazard models were calculated in 10 iterations of fivefold nested cross validation with different feature selection methods (Spearman correlation (spearman)*,* concordance index (concordance), minimum redundancy maximum relevance (mrmr), mutual information feature selection (mifs), and random selection as control) (see Supplemental Figure S1 for a detailed graphical depiction). The manual segmentation was used. Prior to analysis, Yeo-Johnson transformation and z-transformation to mean zero and standard deviation of one were performed. For each iteration of the outer folds, the internal cross validation folds were repeated 11 times to select the median signature size and the top ranking features. The predictive performance in the outer folds was aggregated over all 10 iterations.

We developed models comprising of clinical features (*Clinical*, including the following variables: age, ISUP grade after surgery, initial PSA, maximum PSA prior sRT and rcN status) and radiomic features (*Radiomics*). Finally, a combined *clinical-radiomic* model was generated by using clinical and radiomic features as input into the same pipeline.

### Statistical analysis

Descriptive statistics were performed with Excel 2016 (Microsoft Cooperation, USA). Statistical analysis and model building were performed using R (version 4.1.2, R core team, Vienna, Austria). The primary endpoint of the study was BRFS, defined as time to serum PSA  >0.2 ng/ml above the post-sRT nadir without initiation of additional salvage therapies or death of any cause.

In order to compare the predictive value of the developed models in the test sets within the nested cross validation approach, the following methods were used: time-dependent receiver-operating characteristic (ROC) curves [20], calibration curves (see Supplemental Figure S2) [21], time-dependent discrimination improvement index (tdIDI), time-dependent net reclassification improvement index (tdNRI) (see Supplemental Methods) [22], and a decision curve analysis (DCA) [23]. The median predictor over all 10 × 5 outer testing sets was determined for each patient. Kaplan-Meier analysis [24] was conducted by recording the median value of the predictions in each training set and by applying it as a cut-off value for classification in the respective test sets for all patients. The final classification was determined by majority voting over all 10 iterations.

The C-index and ROC area under the curve (AUC) were calculated as a performance metric. The Wilcoxon rank-sum test was used for comparison of values at a significance level of 0.05.

Decision curve analysis was performed according to Vickers et al. to compare the clinical net benefit of the developed models [25]. Decision curves for “treating no patient” and “treating all patients” were depicted as reference.

## Results

### Patient characteristics

**Fig. 1:**
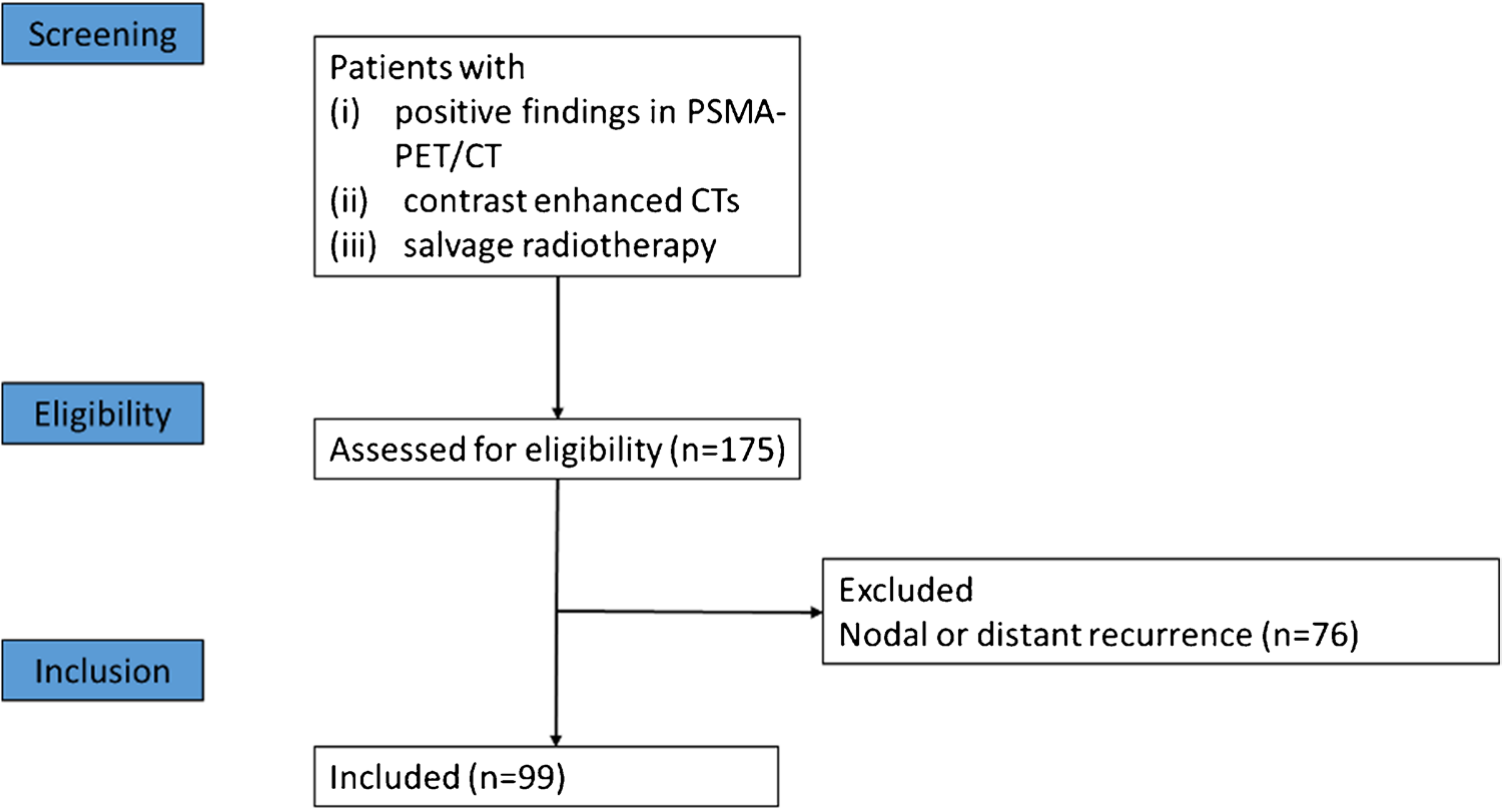
Consort flow diagram. Abbreviations: PSMA-PET/CT = positron-emission tomography targeting prostate-specific membrane antigen combined with computer tomography

Ninety-nine patients with a median follow-up of 29 months (range 3–79 months) were included in this analysis (Fig. 1). No patient died during FU. See Table 1 for details about patient characteristics.

**Table 1:** Patient characteristics

	Site 1		Site 2		Site 3		Overall	
	(*N* = 33)		(*N* = 41)		(*N* = 25)		(*N* = 99)	
Age								
Median [min, max]	74	[49, 83]	68	[50, 82]	78	[58, 87]	72	[49, 87]
ISUP grade after surgery								
1	4	(12.1%)	0	(0%)	2	(8%)	6	(6.1%)
2	10	(30.3%)	2	(4.9%)	8	(32%)	20	(20.2%)
3	6	(18.2%)	24	(58.5%)	9	(36%)	39	(39.4%)
4	7	(21.2%)	6	(14.6%)	4	(16%)	17	(17.2%)
5	6	(18.2%)	9	(22%)	2	(8%)	17	(17.2%)
rcN status								
0	22	(66.7%)	15	(36.6%)	19	(76%)	56	(56.6%)
1	11	(33.3%)	26	(63.4%)	6	(24%)	43	(43.4%)
PSA initial								
Median [min, max]	10	[3.56, 190]	9.73	[1.01, 80]	8.9	[4.24, 43]	9.73	[1.01, 190]
Max PSA before sRT								
Median [min, max]	1.47	[0.26, 10.1]	0.54	[0, 7.74]	2.1	[0.21, 14.8]	1.07	[0, 14.8]
RT dose to prostatic fossa/local recurrence (*α*/*β* = 1.6 Gy)								
< 0 Gy	9	(27.3%)	16	(39.0%)	19	(76.0%)	44	(44.4%)
≥70 Gy	22	(66.7%)	3	(7.3%)	1	(4.0%)	26	(26.3%)
≥72 Gy	2	(6.1%)	19	(46.3%)	5	(20%)	26	(26.3%)
Missing			3	(7.3%)			3	(3.0%)
ADT								
Yes	24	(72.7%)	17	(41.5%)	6	(24%)	47	(47.5%)
No	9	(27.3%)	24	(58.5%)	19	(76%)	52	(52.5%)
Duration of ADT >12 months								
Yes	6	(18.2%)	8	(19.5%)	1	(4%)	15	(15.2%)
No	18	(54.5%)	9	(22%)	5	(20%)	32	(32.3%)
Missing	9	(27.3%)	24	(58.5%)	19	(76%)	52	(52.5%)
Time to biochemical recurrence in months								
Median [min, max]	20	[3, 54]	21	[4, 64]	25	[6, 56]	22	[3, 64]
Biochemical recurrence								
Yes	10	(30.3%)	9	(22%)	7	(28%)	26	(26.3%)
No	23	(69.7%)	32	(78%)	18	(72%)	73	(73.7%)

### Radiomic models outperform clinical models for prediction of biochemical failure

The developed clinical signatures achieved low to moderate performance for prediction of BRFS with a C-index ranging between 0.51 and 0.61 in the test sets. The radiomic signature achieved superior prediction of BRFS with good performance in both training and test set with a C-index ranging between 0.66 and 0.71 in the test set. Combined clinical-radiomic models achieved only moderate performance in the test set with a C-index of 0.60–0.65. The models based on random feature selection performed worse for the radiomic models and similar to other clinical models. See Table 2 for details. Feature reduction with mrmr was chosen for further analyses of all models due to its performance close to the median overall training performance and narrow 95% confidence interval for the clinical and radiomic models (excluding random). For consistency, mrmr was also selected for the combined model.

**Table 2:** Performance of clinical, radiomic, and clinical-radiomic signatures for prediction of biochemical failure after salvage radiotherapy. Results of various feature selection methods are shown

Feature selection method	C-index train (95% CI)	C-index test (95% CI)	Median signature size (min-max)
Clinical signature			
mifs	0.71 (0.60–0.80)	0.51 (0.37–0.64)	1 (1–2)
mrm	0.70 (0.59–0.80)	0.53 (0.40–0.67)	1 (1–2)
spearman	0.64 (0.51–0.77)	0.61 (0.48–0.71)	5 (2–8)
concordance	0.71 (0.62–0.80)	0.51 (0.38–0.62)	1 (1–4)
random	0.68 (0.58–0.78)	0.57 (0.46–0.69)	1 (1–5)
Radiomic signature			
mifs	0.72 (0.61–0.81)	0.71 (0.60–0.81)	1 (1–2)
mrm	0.72 (0.62–0.82)	0.71 (0.57–0.80)	1 (1–2)
spearman	0.81 (0.71–0.88)	0.66 (0.57–0.77)	5 (2–8)
concordance	0.72 (0.59–0.80)	0.71 (0.58–0.81)	1 (1–4)
random	0.82 (0.72–0.89)	0.62 (0.51–0.72)	5 (2–12)
Clinical-radiomic signature			
mifs	0.78 (0.70–0.85)	0.61 (0.48–0.71)	2 (2–6)
mrm	0.78 (0.71–0.85)	0.63 (0.52–0.75)	2 (2–3)
spearman	0.84 (0.78–0.90)	0.65 (0.52–0.75)	5 (1–8)
concordance	0.79 (0.70–0.85)	0.60 (0.47–0.71)	3 (2–5)

**Fig. 2:**
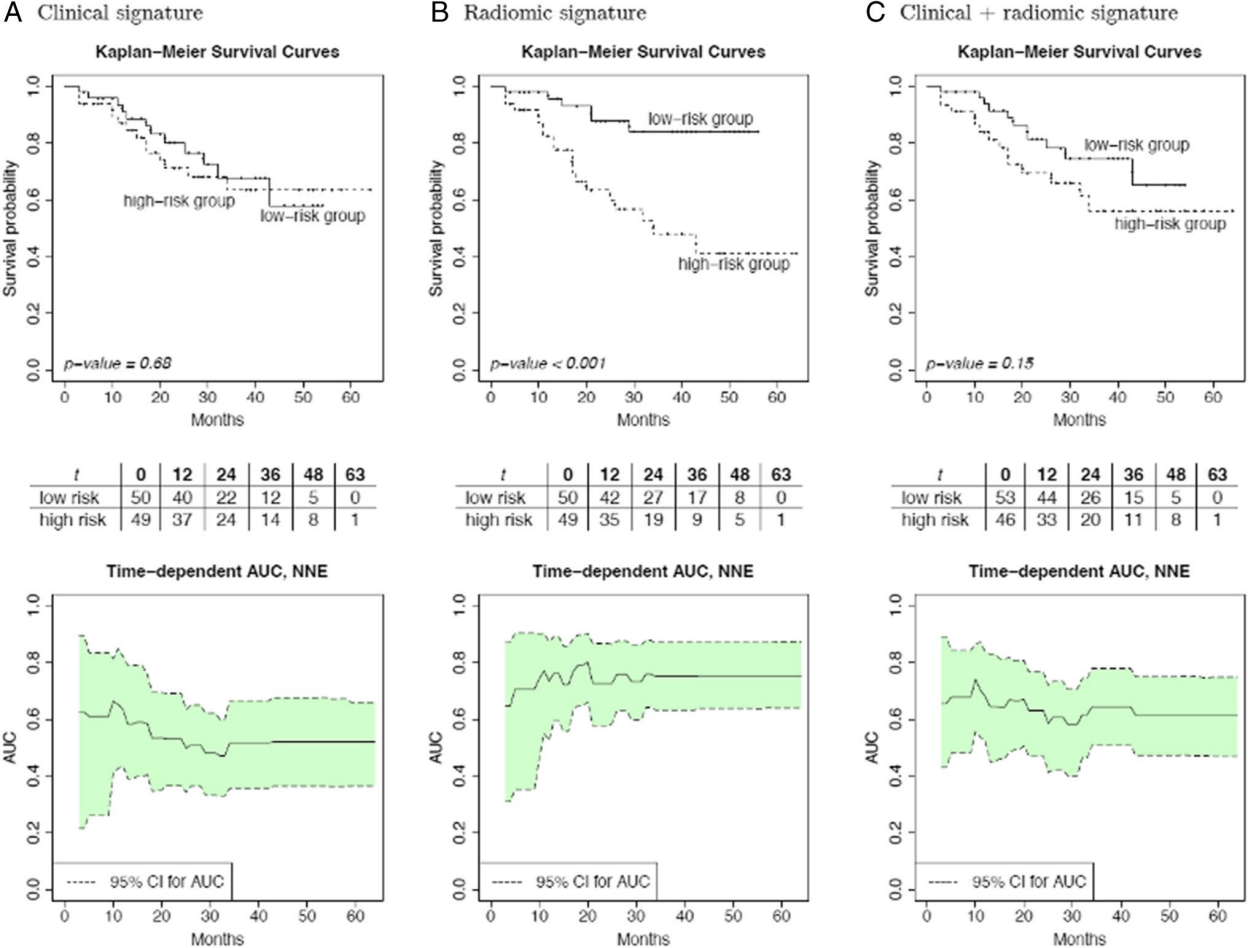
The Kaplan–Meier survival curves and time-dependent area-under-the-curve (AUC) results for the clinical (**A**), radiomic, (**B**) and combined clinical and radiomic (**C**) signatures obtained from repeated nested cross validation

We further stratified patients into low or high probability of BRFS based on predictions of the respective models. Only the radiomic models resulted in significantly different survival probabilities (*p* < 0.001). Time-dependent ROC analysis showed consistent AUC values over time of up to 0.8 for the radiomic signatures up to 60 months of follow-up. Combination of clinical and radiomic signatures showed lower AUCs than the radiomic signature alone. See Fig. 2 for details.

**Fig. 3:**
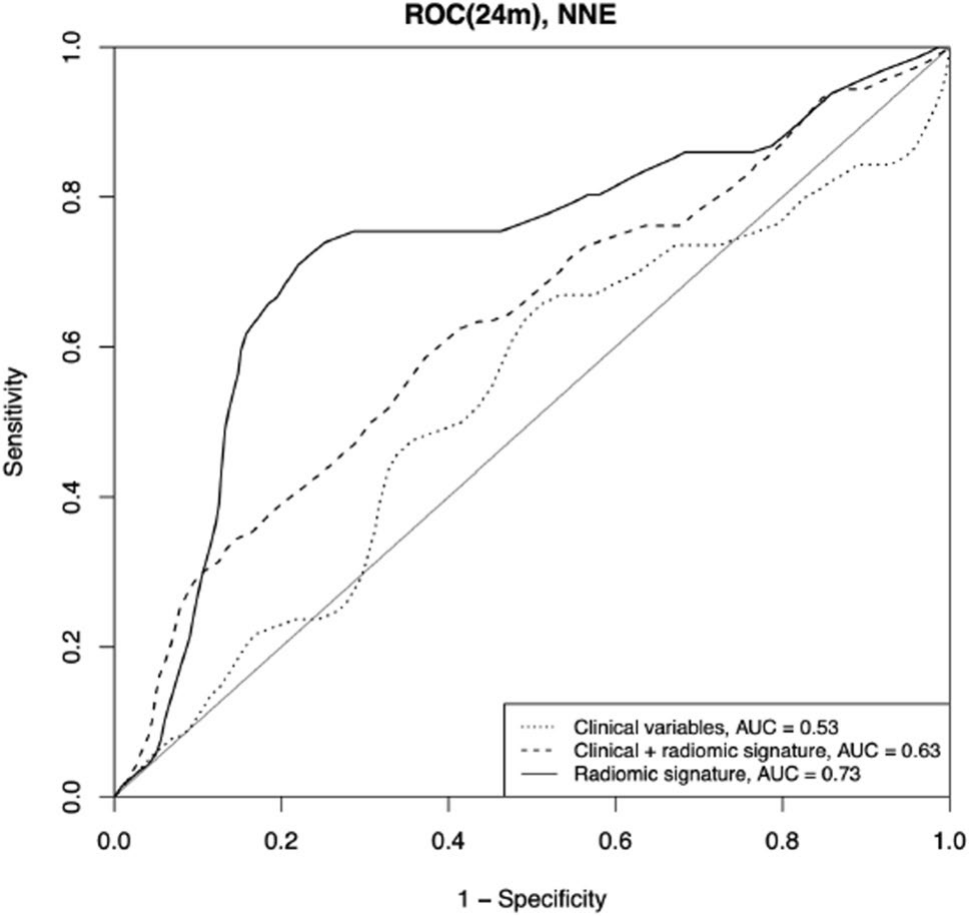
Results of the receiver operator characteristic analysis for the clinical signatures, radiomic signatures, and clinical-radiomic signature at 24 months of follow-up based on repeated nested cross validation (test results in the outer fold) results

At 24-month FU, the clinical signatures, radiomic signatures, and combined clinical and radiomic signatures achieved an AUC of 0.53, 0.73, and 0.63, respectively. See Fig. 3 for details.

### Clinical relevance of the radiomic signature

**Fig. 4:**
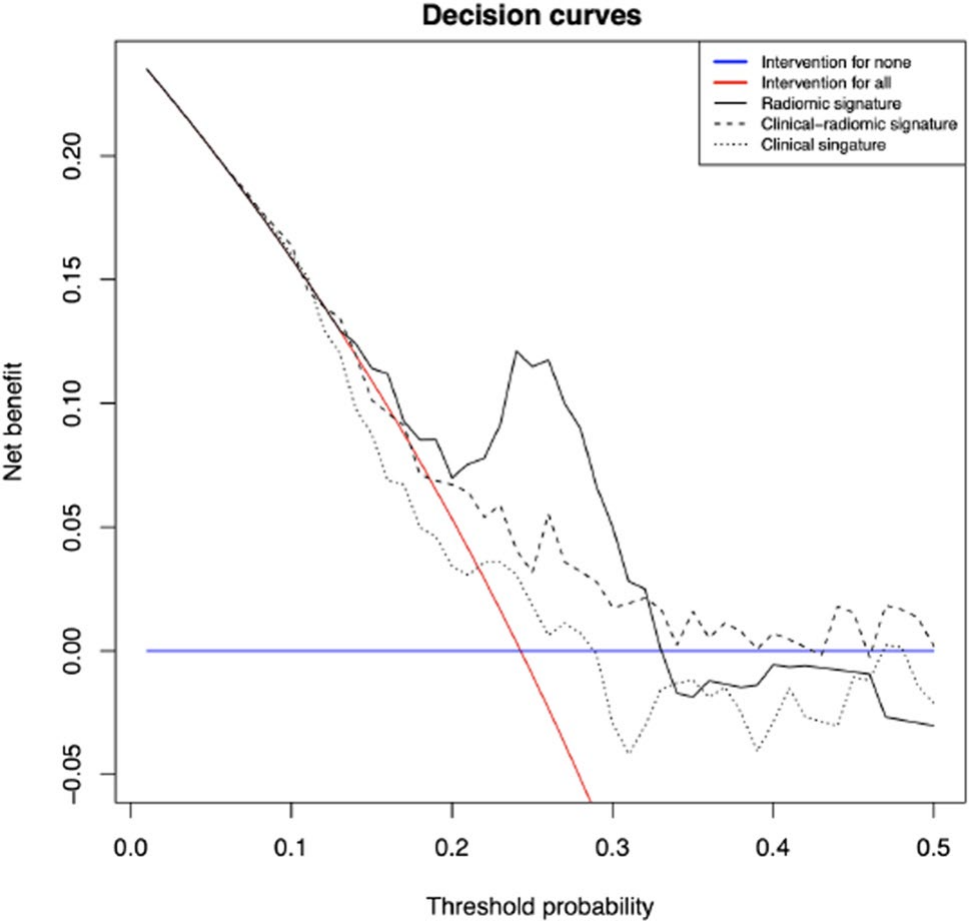
Decision curves for clinical, radiomic, and clinical-radiomic signatures based on repeated nested cross validation results (test sets within the outer folds). Decision curves for “treating no patient” and “treating all patients” were depicted as reference

Decision curve analysis reflects the highest clinical net benefit for the radiomic signatures compared to the two alternative signatures while the combined model also performed better than the clinical model. See Fig. 4 for details.

Despite lower AUC values, the combined clinical-radiomic signatures achieved higher improvements in sensitivity as the radiomic signatures alone compared to the clinical signatures in the test sets of the outer folds (0.78 vs. 0.26). The clinical-radiomic signatures also demonstrated improved tdIDI over the clinical signatures (0.766, *p* = 0.027). The radiomic and clinical-radiomic models achieved a significantly better tdNRI (0.392 and 0.762, respectively) compared to the clinical model (*p* < 0.005) (Table 3).

**Table 3:** shows results of time-dependent reclassification analysis for the clinical, radiomics, and combined clinical-radiomic model based on repeated nested cross validation results (test sets within the outer folds)

Models to compare	Improvement in sensitivitiy	Improvement in specificity	Integrated discrimination improvement index	*p*-value	Net reclassification improvement index	*p*-value
M0: clinical signature M1: radiomic signature	0.263	0.083	0.346	0.221	0.397	<0.005
M0: clinical signature M1: clinical-radiomic signature	0.778	−0.012	0.766	0.027	0.726	<0.005
M0: clinical-radiomic signature M1: radiomic signature	−0.515	0.095	−0.420	0.074	−0.315	<0.005

### CT mean intensity and PSA initial as most important features

For the radiomic signature, the feature “firstorder_mean,” i.e., the mean CT intensity value, was selected as predictive feature for all feature reduction methods with a frequency of 98% in the case for mrmr (see Supplemental Material Table S3 for selected features). For the clinical model, PSA initial was predominantly selected with a frequency of 40% for mrmr. The same two features were the most often selected features in the *clinical-radiomic* model (40% and 23%, respectively). See Table 4 for intensity values stratified by BCR.

**Table 4:** CT intensity values in Hounsfield units stratified by biochemical recurrence (BCR) are shown

	Minimum	1st quartile	Median	Mean	3rd quartile	Max
BCR	−76.54	2.11	18.29	19.82	42.31	86.17
No BCR	−129.38	29.87	46.10	39.39	59.93	110.16

**Fig. 5:**
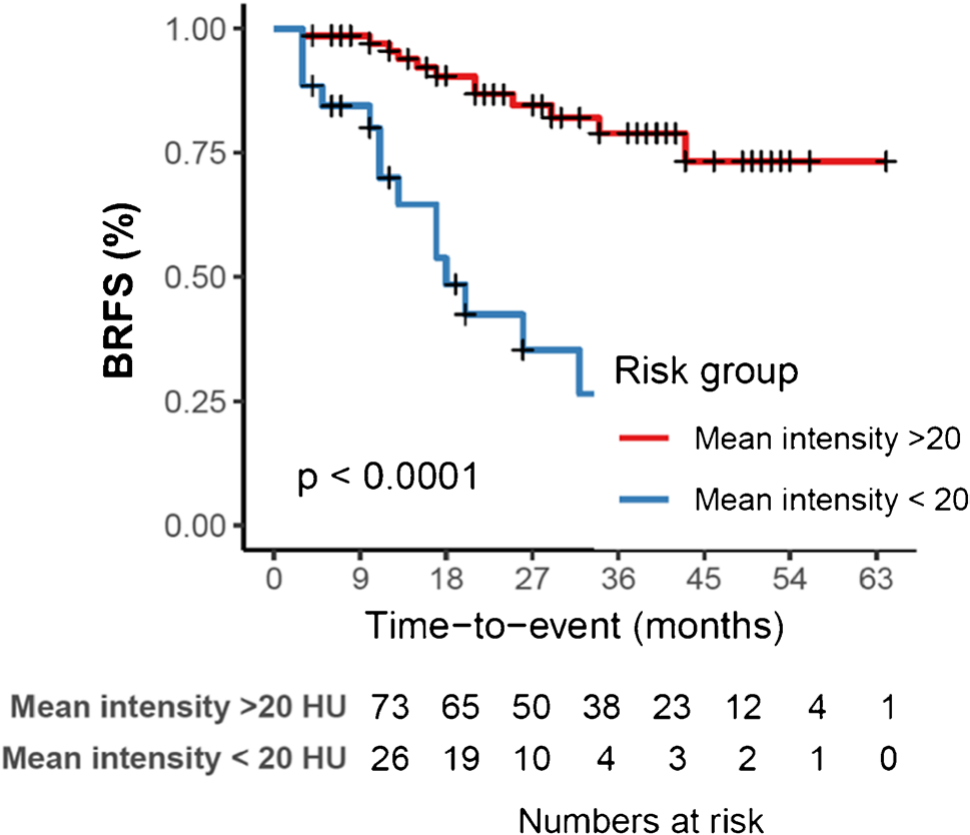
Kaplan-Maier curve for biochemical recurrence free survival (BRFS) stratified after mean intensity of Hounsfield units  <20 and  >20

**Fig. 6:**
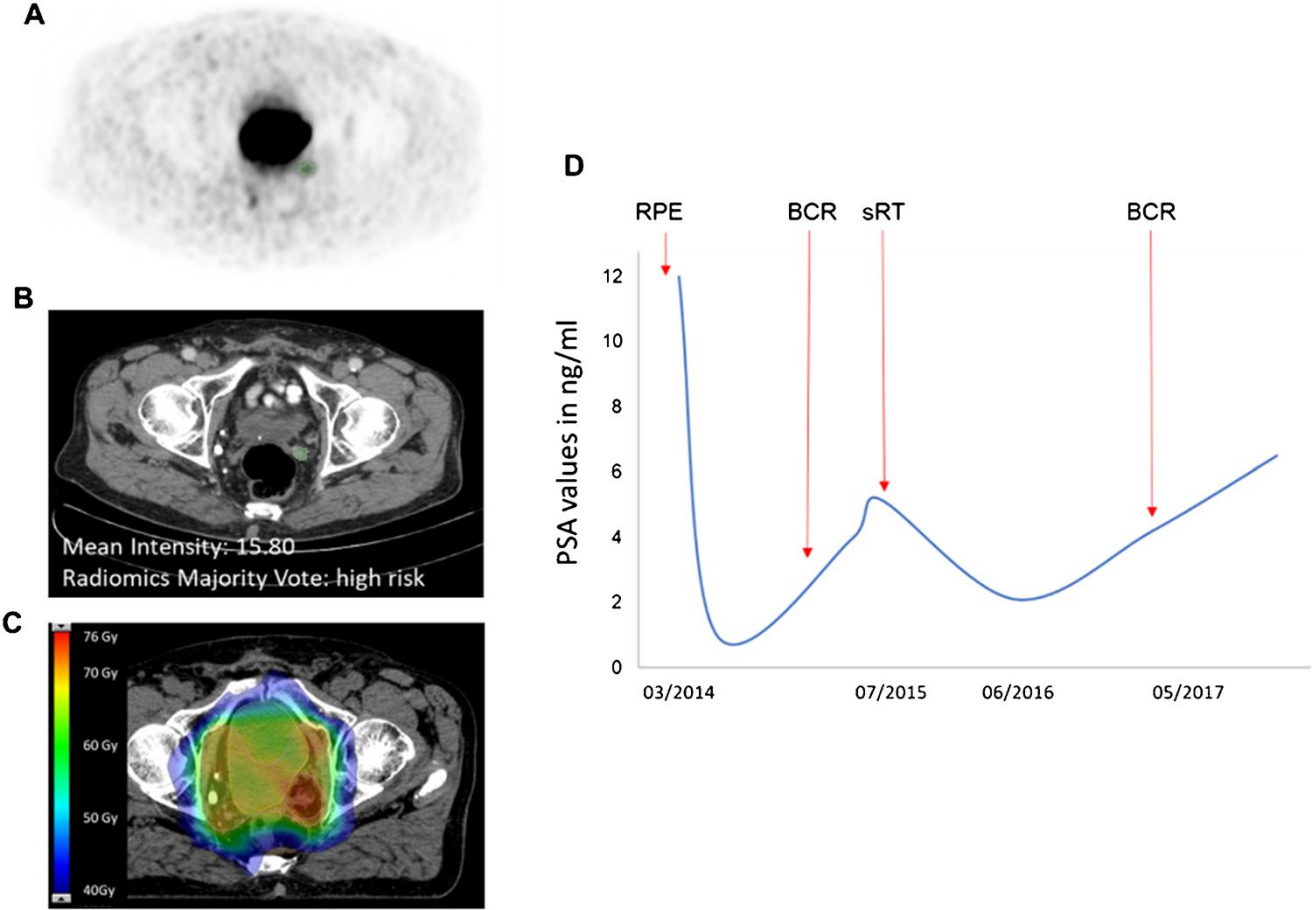
Exemplary patient case: **A** shows the PET-positive local recurrence in the prostatic fossa/seminal vesicle fossa (segmentation green. **B** shows the respective segmentation in the CT scan (segmentation in green). Mean intensity of the segmentation was 15.8 and radiomic majority vote was high risk. **C** shows an axial slide of the radiotherapy plan in colorwash with dose escalation in the area of the local recurrence. **D** shows the prostate-specific antigen (PSA) values of time. Time point of radical prostatectomy (RPE), biochemical recurrence BCR), and salvage radiotherapy (sRT) are highlighted

The maximally selected rank statistics on the complete dataset revealed a mean intensity of 19.7 Hounsfield units (HU) as optimal cut point. A univariate Cox proportional hazard model for CT mean intensity (HR 0.99, *p* = 0.012, Fig. 5) and the respective nomogram is provided in the supplement (Supplemental Material Table S4 and Figure S3). An exemplary patient case is provided in Fig. 6. In a multivariate Cox model including PSA initial (HR 1.01, *p* = 0.052) and CT mean intensity (HR 0.99, *p* = 0.012), only the latter was significant.

## Discussion

In this study, we have developed PSMA-PET-guided CT-based radiomic signatures for prediction of BRFS after sRT due to PCa recurrence using a multicenter cohort from three high-volume centers. We designed the model as a pre-therapeutic tool to guide treatment decision and to identify patients who are at higher or lower risk of relapse and might thus be candidates for treatment intensification or de-intensification. Consequently, treatment-specific parameters, such as delivered radiation dose, were not considered for model building. The developed radiomic signatures yielded good predictive performances and outperformed clinical signatures based on classical histological and clinical parameters. The radiomic model achieved significant patient stratification and demonstrated durable prediction of BRFS in time-dependent ROC analysis. To the best of our knowledge, this is the first study assessing CT-based radiomics in patients who underwent PSMA-PET-based sRT and thus provides novel insights into this field of research.

Analyses of RFs have extensively been performed in primary prostate cancer patients [11]. Most of these studies are based on MRI and demonstrated the ability of radiomics to non-invasively characterize and detect clinically significant PCa and extracapsular extension or predict BCR [26–28].

Fewer studies reported on CT-based radiomics and all of these were performed in the primary setting. Three studies developed CT-based radiomic classifiers with good performance to predict Gleason score and risk groups (AUC 0.70–0.83) [29–31].

Based on PSMA-PET/CT scans, Peeken et al. developed a CT-based model to detect lymph node metastases, which outperformed conventional CT parameters with an AUC of 0.95 in external testing [32], addressing the limited ability of conventional imaging to detect PCa-positive lymph nodes. Acar et al. used CT-based RFs to differentiate between bone metastases and sclerotic areas with good accuracy (AUC 0.76) [33].

Since patients with BCR after surgery experience heterogeneous response rates [4, 34], our study aims to improve risk stratification with commonly available diagnostics for patients receiving sRT based on state-of-the-art diagnostics and to identify patients who might benefit from treatment de-intensification or intensification.

In our study, clinical signatures showed insufficient prognostic value for BRFS after sRT in the test sets, which reflects the deficiency of classical clinical and pathological parameters for prognostication demonstrated by retrospective and prospective studies. However, the developed radiomic signatures outperformed the clinical models with good prognostic values in the test sets. Radiomic signatures and particularly various feature selection methods outperformed clinical models, which demonstrates a certain robustness of these signatures. The inferior performance of the combined clinical and radiomic signatures might be explainable due to the poor prognostic value of clinical parameters and the low patient number for effective model building.

Since no other studies evaluated CT-based radiomics to predict BCR, we cannot directly compare our signatures with other CT-based models. Nevertheless, in comparison with mpMRI-derived RF, the radiomic models in our study performed similarly well with a C-index of  >0.7, considering different clinical scenarios between these studies. DCA demonstrates a net benefit of the radiomic signatures, suggesting that clinical utilization of radiomics can help to identify patients who are at higher risk of BCR after sRT. Whether these patients benefit from intensified treatments and if which kind of treatment intensification is optimal need to be evaluated in future studies.

CT-based radiomics might in future play an even more important role, since technical advantages such as dual-energy CTs provide more image information and may thus allow for more differentiated radiomic analyses. In addition, the prognostic capability of PSMA-based radiomic signatures needs to be evaluated in future studies.

Due to the small patient number, we were not able to separate an external testing cohort, but rather obtained high statistical robustness by applying a nested cross validation approach. Future studies should focus on external validation to demonstrate transferability of models.

The mean intensity within the VOI was selected as the most important RF. Lower intensity values were associated with decreased BRFS. To provide a simple cut-off metric, we applied the maximally selected rank statistics. A cut-off of 19.7 HU was determined as optimal cut-off point for BCR. However, unlimited reduction of HU values is not plausible, since we expect local recurrence to have HU values greater than fat tissue. Thus, this cut-off value should be validated in further studies. Moreover, we provide a univariate Cox model and nomogram trained on the complete cohort for future external validation.

There are several limitations in our study. First we want to mention the retrospective character and possible selection bias. Secondly, we have included patients with LR and NR in this analysis, who experience different outcomes. Separation of both cohorts would have resulted in an insufficient sample size. Nevertheless, development of CT-based radiomic signatures might be influenced to a lesser extent through this heterogeneity in comparison to functional imaging methods. Thirdly, we used an internal validation due to the low number of patients within each institution. However, we applied a sophisticated nested cross validation approach to overcome methodological disadvantages. The inclusion of patients that received ADT may have biased optimal outcome predictions. Again, exclusion of these patients would have significantly reduced the sample number. Finally, the FU in our cohorts is relatively short with a median FU of 29 months.

## Summary

The developed CT-based radiomic signatures outperform clinical/clinical-radiomic signatures for prediction of BCR after sRT and demonstrated durable prediction of biochemical recurrence in time-dependent ROC analysis. Decision curve analysis demonstrates a net benefit for clinical utilization of the radiomic signature. Future studies need to evaluate whether these improved prognostications can be transferred into personalized treatments.

## Supplementary Information

Below is the link to the electronic supplementary material.

Supplementary file1 (DOCX 17 KB)

Supplementary file2 (DOCX 207 KB)

Supplementary file3 (DOCX 30 KB)

## Data Availability

The datasets generated during and/or analyzed during the current study are available from the corresponding author on reasonable request.

## References

[1] Wiegel T, Bartkowiak D, Bottke D, Bronner C, Steiner U, Siegmann A (2014). Adjuvant radiotherapy versus wait-and-see after radical prostatectomy: 10-year follow-up of the ARO 96–02/AUO AP 09/95 trial. Eur Urol.

[2] Stephenson AJ, Shariat SF, Zelefsky MJ, Kattan MW, Butler EB, Teh BS (2004). Salvage radiotherapy for recurrent prostate cancer after radical prostatectomy. JAMA.

[3] Cornford P, van den Bergh RCN, Briers E, Van den Broeck T, Cumberbatch MG, De Santis M, et al. EAU-EANM-ESTRO-ESUR-SIOG guidelines on prostate cancer. Part II-2020 Update: Treatment of Relapsing and Metastatic Prostate Cancer. Eur Urol. 2021;79(2):263–82. 10.1016/j.eururo.2020.09.046 33039206

[4] Tendulkar RD, Agrawal S, Gao T, Efstathiou JA, Pisansky TM, Michalski JM (2016). Contemporary update of a multi-institutional predictive nomogram for salvage radiotherapy after radical prostatectomy. J Clin Oncol.

[5] Fendler WP, Calais J, Eiber M, Flavell RR, Mishoe A, Feng FY (2019). Assessment of 68Ga-PSMA-11 PET accuracy in localizing recurrent prostate cancer: a prospective single-arm clinical trial. JAMA Oncol.

[6] Schmidt-Hegemann NS, Eze C, Li M, Rogowski P, Schaefer C, Stief C (2019). Impact of (68)Ga-PSMA PET/CT on the radiotherapeutic approach to prostate cancer in comparison to CT: a retrospective analysis. J Nucl Med.

[7] Peeken JC, Asadpour R, Specht K, Chen EY, Klymenko O, Akinkuoroye V (2021). MRI-based delta-radiomics predicts pathologic complete response in high-grade soft-tissue sarcoma patients treated with neoadjuvant therapy. Radiother Oncol.

[8] Peeken JC, Neumann J, Asadpour R, Leonhardt Y, Moreira JR, Hippe DS (2021). Prognostic assessment in high-grade soft-tissue sarcoma patients: a comparison of semantic image analysis and radiomics. Cancers.

[9] Peeken JC, Wiestler B, Combs SE, Schober O, Kiessling F, Debus J (2020). Image-guided radiooncology: the potential of radiomics in clinical application. Molecular imaging in oncology.

[10] Zamboglou C, Carles M, Fechter T, Kiefer S, Reichel K, Fassbender TF (2019). Radiomic features from PSMA PET for non-invasive intraprostatic tumor discrimination and characterization in patients with intermediate- and high-risk prostate cancer - a comparison study with histology reference. Theranostics.

[11] Spohn SKB, Bettermann AS, Bamberg F, Benndorf M, Mix M, Nicolay NH (2021). Radiomics in prostate cancer imaging for a personalized treatment approach - current aspects of methodology and a systematic review on validated studies. Theranostics.

[12] Fendler WP, Eiber M, Beheshti M, Bomanji J, Ceci F, Cho S, et al. (68)Ga-PSMA PET/CT: Joint EANM and SNMMI procedure guideline for prostate cancer imaging: version 1.0. Eur J Nucl Med Mol Imaging. 2017;44(6):1014–24. 10.1007/s00259-017-3670-z 28283702

[13] Spohn SKB, Farolfi A, Schandeler S, Vogel MME, Ruf J, Mix M, et al. The maximum standardized uptake value in patients with recurrent or persistent prostate cancer after radical prostatectomy and PSMA-PET-guided salvage radiotherapy-a multicenter retrospective analysis. Eur J Nucl Med Mol Imaging. 2022;50(1):218–27. 10.1007/s00259-022-05931-5 PMC9668780 35984452

[14] Ceci F, Oprea-Lager DE, Emmett L, Adam JA, Bomanji J, Czernin J, et al. E-PSMA: the EANM standardized reporting guidelines v1.0 for PSMA-PET. Eur J Nucl Med Mol Imaging. 2021;48(5):1626–38. 10.1007/s00259-021-05245-y PMC8113168 33604691

[15] Zamboglou C, Fassbender TF, Steffan L, Schiller F, Fechter T, Carles M (2019). Validation of different PSMA-PET/CT-based contouring techniques for intraprostatic tumor definition using histopathology as standard of reference. Radiother Oncol.

[16] van Griethuysen JJM, Fedorov A, Parmar C, Hosny A, Aucoin N, Narayan V (2017). Computational radiomics system to decode the radiographic phenotype. Can Res.

[17] Tixier F, Le Rest CC, Hatt M, Albarghach N, Pradier O, Metges J-P (2011). Intratumor heterogeneity characterized by textural features on baseline <sup>18</sup>F-FDG PET images predicts response to concomitant radiochemotherapy in esophageal cancer. J Nucl Med.

[18] Zwanenburg A, Vallières M, Abdalah MA, Aerts H, Andrearczyk V, Apte A (2020). The image biomarker standardization initiative: standardized quantitative radiomics for high-throughput image-based phenotyping. Radiology.

[19] RabascoMeneghetti A, Zwanenburg A, Leger S, Leger K, Troost EGC, Linge A (2021). Definition and validation of a radiomics signature for loco-regional tumour control in patients with locally advanced head and neck squamous cell carcinoma. Clin Transl Radiat Oncol.

[20] Heagerty PJ, Lumley T, Pepe MS (2000). Time-dependent ROC curves for censored survival data and a diagnostic marker. Biometrics.

[21] Zwanenburg A. familiar: Vignettes and Documentation. 2021.

[22] Liu M, Kapadia AS, Etzel CJ (2010). Evaluating a new risk marker’s predictive contribution in survival models. J Stat Theory Pract.

[23] Sjoberg DD. dcurves: decision curve analysis for model evaluation. 2022.

[24] Simon RM, Subramanian J, Li MC, Menezes S (2011). Using cross-validation to evaluate predictive accuracy of survival risk classifiers based on high-dimensional data. Brief Bioinform.

[25] Vickers AJ, Elkin EB (2006). Decision curve analysis: a novel method for evaluating prediction models. Med Decis Making.

[26] Bourbonne V, Fournier G, Vallières M, Lucia F, Doucet L, Tissot V, et al. External validation of an MRI-derived radiomics model to predict biochemical recurrence after surgery for high-risk prostate cancer. Cancers (Basel). 2020;12(4). 10.3390/cancers12040814 PMC7226108 32231077

[27] Shiradkar R, Ghose S, Jambor I, Taimen P, Ettala O, Purysko AS (2018). Radiomic features from pretreatment biparametric MRI predict prostate cancer biochemical recurrence: preliminary findings. J Magn Reson Imaging.

[28] Zhong QZ, Long LH, Liu A, Li CM, Xiu X, Hou XY (2020). Radiomics of multiparametric MRI to predict biochemical recurrence of localized prostate cancer after radiation therapy. Front Oncol.

[29] Osman SOS, Leijenaar RTH, Cole AJ, Lyons CA, Hounsell AR, Prise KM (2019). Computed tomography-based radiomics for risk stratification in prostate cancer. Int J Radiat Oncol Biol Phys.

[30] Bosetti DG, Ruinelli L, Piliero MA, van der Gaag LC, Pesce GA, Valli M (2020). Cone-beam computed tomography-based radiomics in prostate cancer: a mono-institutional study. Strahlenther Onkol.

[31] Tanadini-Lang S, Bogowicz M, Veit-Haibach P, Huellner M, Pauli C, Shukla V (2018). Exploratory radiomics in computed tomography perfusion of prostate cancer. Anticancer Res.

[32] Peeken JC, Shouman MA, Kroenke M, Rauscher I, Maurer T, Gschwend JE (2020). A CT-based radiomics model to detect prostate cancer lymph node metastases in PSMA radioguided surgery patients. Eur J Nucl Med Mol Imaging.

[33] Acar E, Leblebici A, Ellidokuz BE, Başbınar Y, Kaya G (2019). Machine learning for differentiating metastatic and completely responded sclerotic bone lesion in prostate cancer: a retrospective radiomics study. Br J Radiol.

[34] Zamboglou C, Strouthos I, Sahlmann J, Farolfi A, Serani F, Medici F (2022). Metastasis-free survival and patterns of distant metastatic disease after prostate-specific membrane antigen positron emission tomography (PSMA-PET)-guided salvage radiation therapy in recurrent or persistent prostate cancer after prostatectomy. Int J Radiat Oncol Biol Phys.

