# Supplementary material for: Development of PSMA-PET-guided CT-based radiomic signature to predict biochemical recurrence after salvage radiotherapy
Source: Eur J Nucl Med Mol Imaging. 2023 Mar 16;50(8):2537–47. doi: 10.1007/s00259-023-06195-3 (PMC10250433; doi:10.1007/s00259-023-06195-3)
Supplement: Supplementary file 1 — Supplementary file1 (DOCX 17 KB) [file 259_2023_6195_MOESM1_ESM.docx]

**Supplementary Text**

**Supplementary Methods – Acquisition protocols and scanner types**

Acquisition of CT images was performed according to local protocols: Contrast-enhanced CTs using 120 kVp and exposure of 100–400 mAs (dose modulation) were performed for attenuation correction. Iodine-based contrast agents were used weight adapted and CT scans were performed at portal venous phases approximately 70-80 seconds after injection of contrast agents. Median slice thickness of CT images was 3 mm (range 1.5 – 5) with a median voxel size of 0.97 x 0.97 mm (range 0.68 x 0-68 – 1.17 x 1.17x).

The following scanners were used: UKF: 16-slice Gemini TF Big Bore, 64-slice Gemini TF or Vereos, all Philips Healthcare, USA; TUM: Biograph mCT/128 slice CT, Siemens Healthineers, Germany; LMU: Biograph 64 and Biograph mCT (Siemens Healthineers, Germany) or Discovery 690 (GE Healthcare, USA).

**Supplementary Methods - Modeling Strategy**

Prior to predictive model building, multiple feature reduction steps were applied. The radiomics features space was reduced by applying the following feature reduction methods on the complete data set: First, radiomic feature susceptible to small differences in the segmentation type (manual vs. PET threshold-based) with an intraclass correlation coefficient (ICC 3,1) of less than 0.8 were excluded. Subsequently, the manual segmentation was used. Second, radiomic features that were highly correlated with one of the clinical variables (PSA initial, ISUP, rcN, max PSA) were excluded, i.e., when an absolute value of the Spearman coefficient was equal to or above 0.6. Third, we performed hierarchical clustering with complete linkage and Spearman correlation as a distance metric to link similar radiomic variables together. We cut the tree at height of 0.2. We kept one feature from the cluster as a representative which should have maximum mutual information with the outcome.

The following model steps were performed using 10 iterations of 5-fold nested cross-validation with different feature selection methods and a Cox proportional hazard model (see Supplemental Material for a detailed graphical depiction) as follows: Prior to analysis, all features were processed using Yeo-Johnson transformation and z-transformed to mean zero and standard deviation of one. The transformation parameters obtained in the training folds were applied to the respective test folds of the cross validation. For each iteration of the outer folds, the internal cross validation folds were repeated 11 times with multiple different feature reduction techniques including Spearman correlation (spearman)*,* concordance index (concordance)*,* minimum redundancy maximum relevance (mrmr), and mutual information feature selection (mifs). As control, random features were selected (random). The top five features were recorded for each internal fold and iteration. The features were ranked according to their frequency of recording. The signature size “k” was chosen as the median signature size over all 11 iterations of the internal folds. Finally, the “*k*” top ranking features were selected as an input for multivariate cox proportional hazard models in the respective outer folds. The predictive performance of the training and test sets in the outer folds were aggregated over all 10 iterations for all feature reduction strategies.

The best feature reduction strategy for further analysis was chosen as closest to the median performance over all models on the respective training sets of the outer fold and with the smallest 95% CI if multiple had the same C-index.

**Supplementary Methods – Cross-validation**

Cross validation was performed according to the following scheme:

***Calibration plot***

The Cox model assumes that the probability that a patient survives past time *t* is given by

${\text{S}_{\text{0}}\text{(t)}}^{\text{e}^{\text{b}_{\text{i}}\text{X}_{\text{i}}}}$,

where *X_i_* are independent variables, *b_i_* are learnt by model. The *S_0_()* is not learnt by the model, we use the Kaplan Meier method to estimate it and thus obtain predicted survival probabilities.

Averaged by groups: Compute statistics 20 times and average their values. Statistics are calculated in each group. Groups are formed in the randomized way

Bias-corrected: Compute statistics from the bootstrap samples and average their values by groups. Number of bootstrap samples is 400. Statistics are calculated in each group. Groups are formed in the randomized way

Quantile-based: Form groups so that there is an approximately equal number of patients within each group. Compute statistics in each group

Spline fit: Fit the adaptive hazard regression to predicted survival probabilities

***Time-depenent IDI: Adopting Liu et al. approach to the scores greater than one***

We will follow the authors’ notation.

By definition, $IS(t)$ is the integral of sensitivity over all possible cut-off values and $IP(t)$ is the corresponding integral of ‘one minus specificity’. Let $u$ be cut-off values for $Z$. Liu et al. restrict the variable $Z$ to the interval $[0, 1]$. However, the estimators introduced by them are also valid for $Z \in[0, +\infty)$. Let $u \in\left[ 0, +\infty\right)$ and $f$ be the probability density function of $Z$

$$IS(t)=\int_{0}^{+\infty} \int_{u}^{+\infty} f\left( z | D\left( t \right)=1 \right)dzdu, IP(t)=\int_{0}^{+\infty} \int_{u}^{+\infty} f\left( z | D(t)=0 \right)dzdu$$

Interchanging the order of integration, we get

$$IS(t)=\int_{0}^{+\infty} \int_{0}^{z} f\left( z | D(t)=1 \right)dudz= \int_{0}^{+\infty} zf\left( z | D(t)=1 \right)dz,$$

$$IP(t)=\int_{0}^{+\infty} \int_{0}^{z} f\left( z | D(t)=0 \right)dudz= \int_{0}^{+\infty} zf\left( z | D(t)=0 \right)dz$$

Liu et al. rewrote the conditional density functions of risk scores $Z$as

$$f\left( z | D(t)=1 \right)= \frac{\left( 1-S\left( t | z \right) \right) \times f(z)}{\mathbb{E}(1-S\left( t | Z \right))}, f\left( z | D\left( t \right)=0 \right)= \frac{S\left( t | z \right) \times f\left( z \right)}{\mathbb{E}\left( S\left( t | Z \right) \right)}$$

They do not change their form under new domain of $Z$. Substituting them in the expressions for $IS\left( t \right)$ and $IP(t)$ we will get the result equal to the original definition

$$IS\left( t \right)=\int_{0}^{+\infty} z\times\frac{\left( 1-S\left( t | z \right) \right) \times f(z)}{\mathbb{E}(1-S\left( t | Z \right))}dz=\frac{\mathbb{E(}Z\left( 1-S(t|Z \right))}{\mathbb{E}(1-S\left( t | Z \right))}= \frac{\mathbb{E}\left( Z \right)\mathbb{-E}\left( Z\times S(t|Z \right))}{\mathbb{E}(1- S\left( t | Z \right))}$$

and

$$IP\left( t \right)=\int_{0}^{+\infty} z\times\frac{S\left( t | z \right) \times f\left( z \right)}{\mathbb{E}\left( S\left( t | Z \right) \right)}dz=\frac{\mathbb{E}\left( Z\times S\left( t | Z \right) \right)}{\mathbb{E}\left( S\left( t | Z \right) \right)}$$
