# Supplementary material for: Development of PSMA-PET-guided CT-based radiomic signature to predict biochemical recurrence after salvage radiotherapy
Source: Eur J Nucl Med Mol Imaging. 2023 Mar 16;50(8):2537–47. doi: 10.1007/s00259-023-06195-3 (PMC10250433; doi:10.1007/s00259-023-06195-3)
Supplement: Supplementary file 2 — Supplementary file2 (DOCX 207 KB) [file 259_2023_6195_MOESM2_ESM.docx]

**Supplementary Figures**

## **Figure S1: Graphical Depcition of Cross Validation**

##
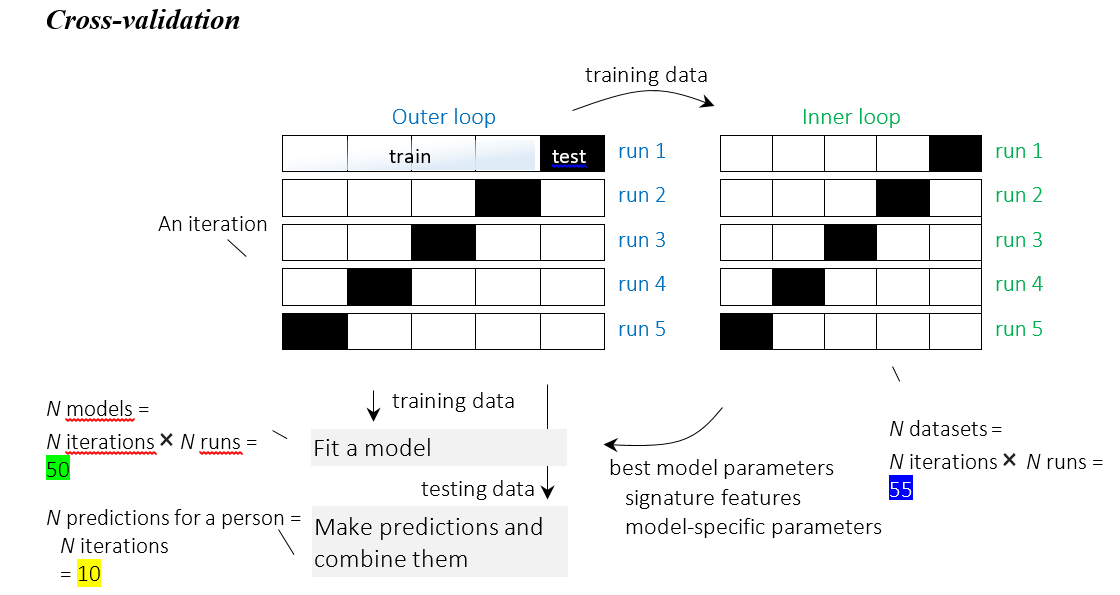


**Figure S2: Calibration Curves**


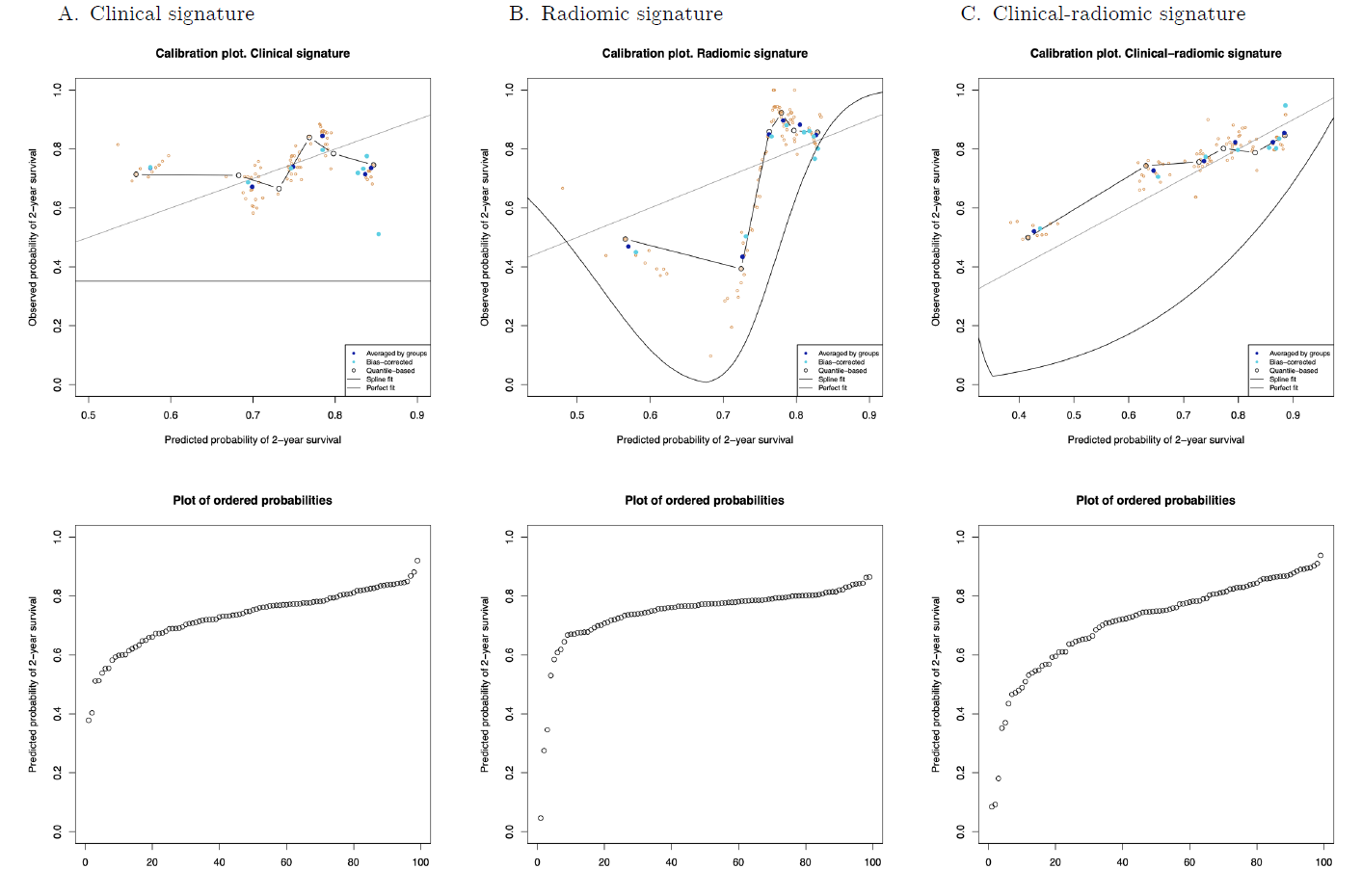


## **Figure S3: Nomogram for CT mean intensity calculated on the complete dataset for 24 month freedom from biochemical failure (FFBF).**
