# Supplementary material for: Development of PSMA-PET-guided CT-based radiomic signature to predict biochemical recurrence after salvage radiotherapy
Source: Eur J Nucl Med Mol Imaging. 2023 Mar 16;50(8):2537–47. doi: 10.1007/s00259-023-06195-3 (PMC10250433; doi:10.1007/s00259-023-06195-3)
Supplement: Supplementary file 3 — Supplementary file3 (DOCX 30 KB) [file 259_2023_6195_MOESM3_ESM.docx]

**Supplementary Tables**

**Table S1: Salvage RT concepts**

| **Center** | **Dose to fossa (EQD2, α/β=1.6 Gy)** | **Indication (and field) for sRT to elective pelvic lymphatics** | **Dose to elective pelvic lymphatics and PET positive pelvic LN** | **Indication (and duration) of androgen deprivation therapy** |
| --- | --- | --- | --- | --- |
| Freiburg | Dose to fossa (R0): 64 Gy  Dose to fossa (R1): 66-68 Gy  Dose to local recurrence in PET: 68-72 Gy | pN+ status in surgery (whole-pelvis)  Positive pelvic LNs in PSMA PET/CT (whole-pelvis) | Elective pelvic lymphatics: 42.5-47.6 Gy  PET positive pelvic LN 50-60 Gy | pN+ status (0-24 months)  PSA prior to sRT >0.7 ng/ml (6 months)  Positive pelvic LNs in PET (6-24 months)  + individual decision (e.g depending on comorbidities) |
| Munich LMU | Dose to fossa: 66 Gy  (in case of whole pelvis RT: 64.75 Gy)  Dose to local recurrence: 70 Gy (in case of whole pelvis RT: 72.2 Gy) | pN+ status in surgery (whole- pelvis)  positive pelvic LNs in PSMA PET/CT (whole-pelvis) | Elective pelvic lymphatics: 46.75 Gy  PET-positive LNs: 64.75 Gy | Positive pelvic LNs in PET (6-24 months)  positive local recurrence in PET (6-24 months)  PSA prior to sRT > 0.7 ng/ml and Gleason 8-10 (6-24 months)  + individual decision (depending on comorbidities, Gleason, PSA prior to sRT) |
| Munich TUM | Dose to fossa: 68 Gy  Dose to local recurrence in PET: 82.88 Gy (SIB, 76.5 Gy in fractions of 2.25 Gy) | pN+ status in surgery, lymph node dissection with <10 lymph nodes, or risk for lymph node involvement of ≥20% (whole-pelvis)  Positive pelvic LNs in PSMA PET/CT (whole-pelvis) | Elective pelvic lymphatics: 47.04 Gy (50.4 Gy in fractions of 1.8 Gy)  PET positive pelvic LN: 60.76 Gy (SIB, 58.8 Gy in fractions of 2.1 G) or 65.71 Gy (SIB, 61.6 Gy in fractions of 2.2 Gy) | PSA prior to sRT >0.7 ng/ml (6-24 months)  + individual decision (tumor conference) |

**Table S2: Extracted radiomics features (n=104)**

All extracted features were computed according the “image biomarker standardization initiative” (IBSI) guidelines [1].

|  | **Shape Features** |
| --- | --- |
| 1.) | Mesh Volume |
| 2.) | Voxel Volume |
| 3.) | Surface Area |
| 4.) | Surface Volume Ratio |
| 5.) | Sphericity |
| 6.) | Maximum 3D Diameter |
| 7.) | Maximum 2D Diameter Slice |
| 8.) | Maximum 2D Diameter Column |
| 9.) | Maximum 2D Diameter Row |
| 10.) | Major Axis |
| 11.) | Minor Axis |
| 12.) | Least Axis |
| 13.) | Elongation |
| 14.) | Flatness |
|  | **First Order Features** |
| 1.) | Energy |
| 2.) | Intensity Histogram Entropy |
| 3.) | Minimum |
| 4.) | 10th Percentile |
| 5.) | 90th Percentile |
| 6.) | Maximum |
| 7.) | Mean |
| 8.) | Median |
| 9.) | Interquartile Range |
| 10.) | Range |
| 11.) | Mean Absolute Deviation (MAD) |
| 12.) | Root Mean Squared (RMS) |
| 13.) | Skewness |
| 14.) | Excess Kurtosis |
| 15.) | Variance |
| 16.) | Intensity Histogram Uniformity |
|  | **Gray Level Co-occurrence Matrix (GLCM) Features** |
| 1.) | Autocorrelation |
| 2.) | Joint Average |
| 3.) | Cluster Prominence |
| 4.) | Cluster Shade |
| 5.) | Cluster Tendency |
| 6.) | Contrast |
| 7.) | Correlation |
| 8.) | Difference Average |
| 9.) | Difference Entropy |
| 10.) | Difference Variance |
| 11.) | Joint Energy (IBSI: Angular Second Moment) |
| 12.) | Joint Entropy |
| 13.) | Informal Measure of Correlation (IMC) 1 |
| 14.) | Informal Measure of Correlation (IMC) 2 |
| 15.) | Inverse Difference Moment (IDM) |
| 16.) | Inverse Difference Moment Normalized (IDMN) |
| 17.) | Inverse Difference (ID) |
| 18.) | Inverse Difference Normalized (IDN) |
| 19.) | Inverse Variance |
| 20.) | Maximum Probability (IBSI: Joint maximum) |
| 21.) | Sum Entropy |
| 22.) | Sum of Squares (IBSI: Sum of Squares) |
| 23.) | Maximal Correlation Coefficient (MCC) |
|  | **Gray Level Size Zone Matrix (GLSZM) Features** |
| 1.) | Small Area Emphasis (SAE) |
| 2.) | Large Area Emphasis (LAE) |
| 3.) | Gray Level Non-Uniformity (GLN) |
| 4.) | Gray Level Non-Uniformity Normalized (GLNN) |
| 5.) | Size-Zone Non-Uniformity (SZN) |
| 6.) | Size-Zone Non-Uniformity Normalized (SZNN) |
| 7.) | Zone Percentage (ZP) |
| 8.) | Gray Level Variance (GLV) |
| 9.) | Zone Variance (ZV) |
| 10.) | Zone Entropy (ZE) |
| 11.) | Low Gray Level Zone Emphasis (LGLZE) |
| 12.) | High Gray Level Zone Emphasis (HGLZE) |
| 13.) | Small Area Low Gray Level Emphasis (SALGLE) |
| 14.) | Small Area High Gray Level Emphasis (SAHGLE) |
| 15.) | Large Area Low Gray Level Emphasis (LALGLE) |
| 16.) | Large Area High Gray Level Emphasis (LAHGLE) |
|  | **Gray Level Run Length Matrix (GLRLM) Features** |
| 1.) | Short Run Emphasis (SRE) |
| 2.) | Long Run Emphasis (LRE) |
| 3.) | Gray Level Non-Uniformity (GLN) |
| 4.) | Gray Level Non-Uniformity Normalized (GLNN) |
| 5.) | Run Length Non-Uniformity (RLN) |
| 6.) | Run Length Non-Uniformity Normalized (RLNN) |
| 7.) | Run Percentage (RP) |
| 8.) | Gray Level Variance (GLV) |
| 9.) | Run Variance (RV) |
| 10.) | Run Entropy (RE) |
| 11.) | Low Gray Level Run Emphasis (LGLRE) |
| 12.) | High Gray Level Run Emphasis (HGLRE) |
| 13.) | Short Run Low Gray Level Emphasis (SRLGLE) |
| 14.) | Short Run High Gray Level Emphasis (SRHGLE) |
| 15.) | Long Run Low Gray Level Emphasis (LRLGLE) |
| 16.) | Long Run High Gray Level Emphasis (LRHGLE) |
|  | **Neighbouring Gray Tone Difference Matrix (NGTDM) Features** |
| 1.) | Coarseness |
| 2.) | Contrast |
| 3.) | Busyness |
| 4.) | Complexity |
| 5.) | Strength |
|  | **Gray Level Dependence Matrix (GLDM) Features** |
| 1.) | Small Dependence Emphasis (SDE) |
| 2.) | Large Dependence Emphasis (LDE) |
| 3.) | Gray Level Non-Uniformity (GLN) |
| 4.) | Dependence Non-Uniformity (DN) |
| 5.) | Dependence Non-Uniformity Normalized (DNN) |
| 6.) | Gray Level Variance (GLV) |
| 7.) | Dependence Variance (DV) |
| 8.) | Dependence Entropy (DE) |
| 9.) | Low Gray Level Emphasis (LGLE) |
| 10.) | High Gray Level Emphasis (HGLE) |
| 11.) | Small Dependence Low Gray Level Emphasis (SDLGLE) |
| 12.) | Small Dependence High Gray Level Emphasis (SDHGLE) |
| 13.) | Large Dependence Low Gray Level Emphasis (LDLGLE) |
| 14.) | Large Dependence High Gray Level Emphasis (LDHGLE) |

[1] Zwanenburg A, Leger S, Vallières M, Löck S. Image biomarker standardisation initiative. ArXiv 2016;1612.07003.

**Table S3: Selected features**

The relative frequency of feature selection for all three models is listed here. The most frequent n features per feature selection method are printed in bold whereby n presents the number of selected features per model (Radiomics: 1, Clinical: 1, Clincal-Radiomics: 2).

| **Feature Selection Method** | **Feature Name** | **Relative Frequency of Selection** |
| --- | --- | --- |
| ***Radiomics*** | | |
| **concordance** | **original_firstorder_Mean** | **0.68** |
| concordance | original_firstorder_Median | 0.15 |
| concordance | original_glcm_ClusterShade | 0.11 |
| concordance | original_firstorder_RootMeanSquared | 0.04 |
| concordance | original_glcm_Imc2 | 0.01 |
| concordance | original_glcm_MCC | 0.01 |
| **mifs** | **original_firstorder_Mean** | **0.94** |
| mifs | original_glcm_DifferenceEntropy | 0.02 |
| mifs | original_glcm_Imc2 | 0.02 |
| mifs | original_shape_MajorAxisLength | 0.02 |
| **mrmr** | **original_firstorder_Mean** | **0.98** |
| mrmr | original_shape_MajorAxisLength | 0.02 |
| **random** | **original_firstorder_Mean** | **0.13** |
| random | original_firstorder_RootMeanSquared | 0.12 |
| random | original_firstorder_Median | 0.12 |
| random | original_glcm_MCC | 0.12 |
| random | original_glcm_Imc2 | 0.08 |
| random | original_gldm_LargeDependenceLowGrayLevelEmphasis | 0.07 |
| random | original_glrlm_RunVariance | 0.06 |
| random | original_ngtdm_Contrast | 0.04 |
| random | original_glcm_DifferenceEntropy | 0.05 |
| random | original_firstorder_InterquartileRange | 0.05 |
| random | original_shape_MajorAxisLength | 0.04 |
| random | original_ngtdm_Busyness | 0.03 |
| random | original_glcm_ClusterShade | 0.1 |
| **spearman** | **original_firstorder_Mean** | **0.17** |
| spearman | original_glcm_ClusterShade | 0.16 |
| spearman | original_glrlm_RunVariance | 0.16 |
| spearman | original_firstorder_RootMeanSquared | 0.11 |
| spearman | original_firstorder_Median | 0.1 |
| spearman | original_ngtdm_Contrast | 0.1 |
| spearman | original_glcm_MCC | 0.06 |
| spearman | original_glcm_Imc2 | 0.03 |
| spearman | original_shape_MajorAxisLength | 0.03 |
| spearman | original_firstorder_InterquartileRange | 0.02 |
| spearman | original_gldm_LargeDependenceLowGrayLevelEmphasis | 0.02 |
| spearman | original_glcm_DifferenceEntropy | 0.02 |
| spearman | original_ngtdm_Busyness | 0.01 |
| ***Clinical*** | | |
| **concordance** | **PSA_initial** | **0.49** |
| concordance | max_PSA_before_sRT | 0.43 |
| concordance | rcN_Status | 0.08 |
| **mifs** | **PSA_initial** | **0.41** |
| mifs | max_PSA_before_sRT | 0.39 |
| mifs | rcN_Status | 0.19 |
| mifs | Gleason_Score_after_Surgery | 0.01 |
| **mrmr** | **PSA_initial** | **0.4** |
| mrmr | max_PSA_before_sRT | 0.38 |
| mrmr | rcN_Status | 0.21 |
| mrmr | Gleason_Score_after_Surgery | 0.01 |
| **random** | **Age** | **0.25** |
| random | PSA_initial | 0.23 |
| random | max_PSA_before_sRT | 0.2 |
| random | Gleason_Score_after_Surgery | 0.17 |
| random | rcN_Status | 0.15 |
| **spearman** | **rcN_Status** | **0.37** |
| spearman | PSA_initial | 0.25 |
| spearman | Gleason_Score_after_Surgery | 0.22 |
| spearman | max_PSA_before_sRT | 0.16 |
| **Clinical + Radiomics** | | |
| **concordance** | **original_firstorder_Mean** | **0.33** |
| **concordance** | **PSA_initial** | **0.17** |
| concordance | max_PSA_before_sRT | 0.15 |
| concordance | original_firstorder_Median | 0.15 |
| concordance | original_glcm_ClusterShade | 0.1 |
| concordance | original_firstorder_RootMeanSquared | 0.07 |
| concordance | rcN_Status | 0.03 |
| concordance | original_shape_MajorAxisLength | 0.01 |
| **mifs** | **original_firstorder_Mean** | **0.4** |
| **mifs** | **PSA_initial** | **0.23** |
| mifs | max_PSA_before_sRT | 0.21 |
| mifs | rcN_Status | 0.1 |
| mifs | original_firstorder_RootMeanSquared | 0.02 |
| mifs | Gleason_Score_after_Surgery | 0.01 |
| mifs | original_firstorder_Median | 0.01 |
| mifs | original_glcm_Imc2 | 0.01 |
| mifs | original_glrlm_RunVariance | 0.01 |
| **mrmr** | **original_firstorder_Mean** | **0.41** |
| **mrmr** | **PSA_initial** | **0.24** |
| mrmr | max_PSA_before_sRT | 0.22 |
| mrmr | rcN_Status | 0.12 |
| mrmr | Gleason_Score_after_Surgery | 0.01 |
| mrmr | original_firstorder_RootMeanSquared | 0.01 |
| **spearman** | **original_firstorder_Mean** | **0.13** |
| **spearman** | **original_glcm_ClusterShade** | **0.12** |
| spearman | original_glrlm_RunVariance | 0.11 |
| spearman | rcN_Status | 0.11 |
| spearman | original_firstorder_Median | 0.09 |
| spearman | original_firstorder_RootMeanSquared | 0.09 |
| spearman | PSA_initial | 0.07 |
| spearman | original_glcm_MCC | 0.07 |
| spearman | Gleason_Score_after_Surgery | 0.06 |
| spearman | max_PSA_before_sRT | 0.05 |
| spearman | original_ngtdm_Contrast | 0.04 |
| spearman | original_glcm_Imc2 | 0.02 |
| spearman | original_shape_MajorAxisLength | 0.02 |
| spearman | original_glcm_DifferenceEntropy | 0.01 |

**Table S4: Cox proportional hazard model for CT mean intensity calculated on the complete dataset for freedom from biochemical failure (FFBF)**

| **Variable** | **Coefficient (standard error)** | **HR (95 % Confidence Interval)** | **Concordance** | **P Value** |
| --- | --- | --- | --- | --- |
| **Original_firstorder_Mean** | **-0.0090 (0.0036)** | **0.991 (0.984 – 0. 998)** | **0.71** | **0.013** |
